# Regulation of semen quality by fatty acids in diets, extender, and semen

**DOI:** 10.3389/fvets.2023.1119153

**Published:** 2023-04-27

**Authors:** Chongshan Yuan, Jun Wang, Wenfa Lu

**Affiliations:** ^1^Joint Laboratory of the Modern Agricultural Technology International Cooperation, Ministry of Education, Jilin Agricultural University, Changchun, China; ^2^Key Lab of the Animal Production, Product Quality, and Security, Ministry of Education, Jilin Agricultural University, Changchun, China

**Keywords:** fatty acids, semen, diets, extender, sperm

## Abstract

Fatty acids (FAs) are classified into different types according to the degree of hydrocarbon chain saturation, including saturated fatty acids (SFAs), monounsaturated fatty acids (MUFAs), omega-3 polyunsaturated fatty acids (omega-3 PUFAs) and omega-6 polyunsaturated fatty acids (omega-6 PUFAs), which play an important role in maintaining semen quality. This review focuses on the regulation of FAs in semen, diet and extender on semen quality, and expounds its effects on sperm motility, plasma membrane integrity, DNA integrity, hormone content, and antioxidant capacity. It can be concluded that there are species differences in the FAs profile and requirements in sperm, and their ability to regulate semen quality is also affected by the addition methods or dosages. Future research directions should focus on analyzing the FAs profiles of different species or different periods of the same species and exploring suitable addition methods, doses and mechanism of regulating semen quality.

## 1. Introduction

**Figure 1:**
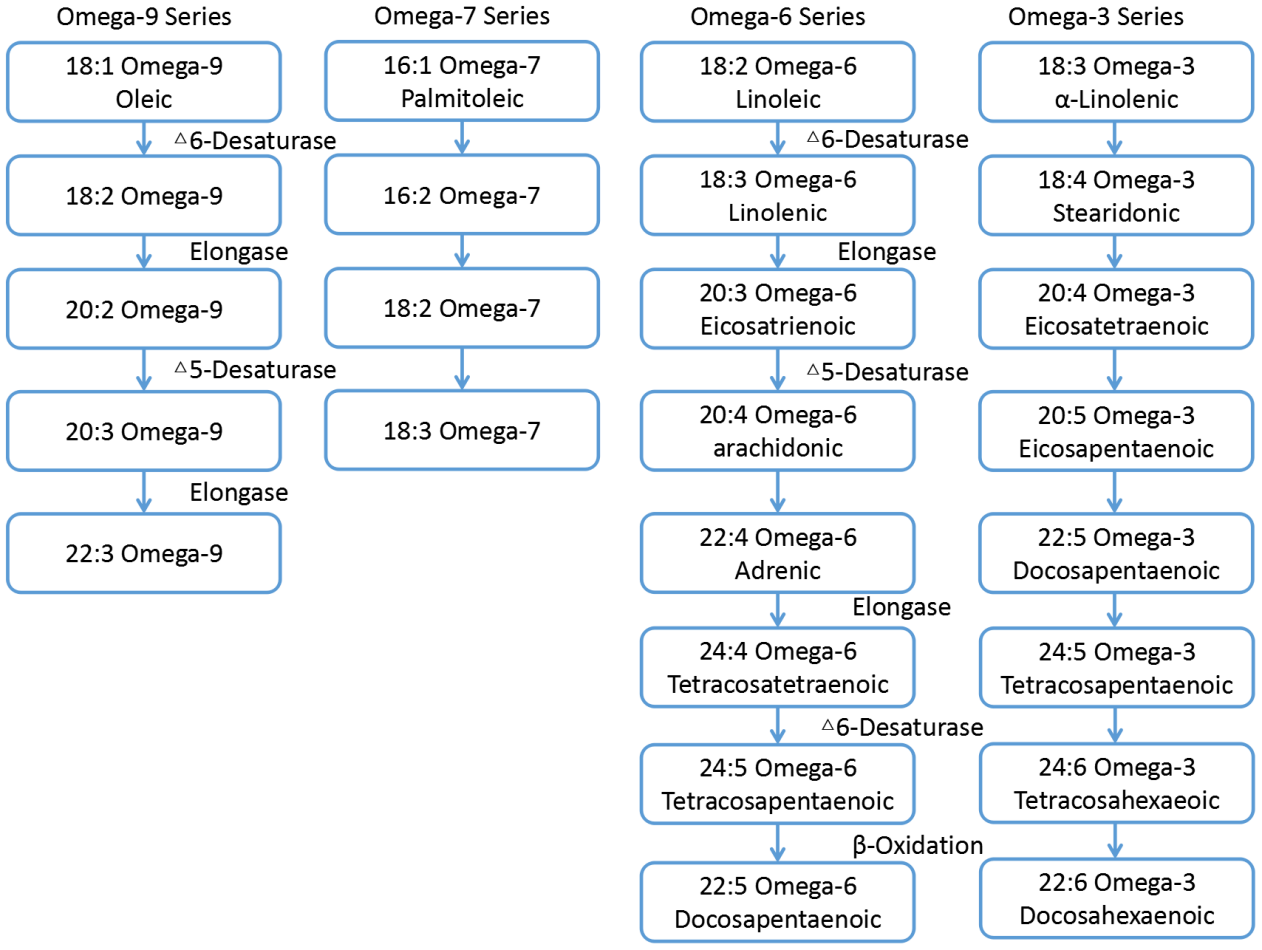
Schematic pathways of various fatty acids metabolism.

Fatty acids (FAs) is the main component of dietary fat. Animals cannot synthesize FAs due to the lack of related desaturases and elongases (1). Therefore, these animals must obtain FAs or their precursors from the diet (2), as they are essential for many processes including growth, reproduction, vision, and brain development (3). The synthetic pathway of various FAs are shown in Figure 1 (4). It can be seen that the appropriate intake of FAs in the diet plays an important role in maintaining its composition in sperm and the reproductive ability (5). omega-3 polyunsaturated fatty acids (omega-3 PUFAs) and omega-6 polyunsaturated fatty acids (omega-6 PUFAs) are essential for multiple functions in the body, including synthesis of prostaglandins, leukotrienes, cell membranes, phospholipids, retinal photoreceptors (vision), gray matter (brain tissue), testes, and sperm (6). It is also reported that dietary PUFAs could regulate steroid hormone secretion (7).

FAs are important in male sperm because they are associated with membrane fluidity, acrosome reaction, sperm motility and viability (8). FAs in sperm membranes play a major role in sperm structure and function (9), and are required to facilitate membrane fusion events associated with fertilization (10, 11). Among them, PUFAs can penetrate the sperm cell membrane, improve the scalability of the sperm plasma membrane, maintain its structural and functional integrity, enhance the osmotic resistance of the acrosome membrane, and provide protection against physiological or thermal changes during cryopreservation (12, 13). Numerous studies have reported that the addition of appropriate levels of PUFAs to semen extender could improve sperm antioxidant capacity and DNA integrity (14), and reduce oxidative stress levels (15).

On the other hand, the composition of FAs in sperm may vary by species. In stallion, the sperm contains high levels of docosapentaenoic acid (DPA), representing on average the 49.9%, followed by palmitic acid (PA) and stearic acid (SA), representing the 17.6 and 8.7%, respectively, (16). The predominant FAs of dog seminal plasma were C16: 0 (30.4%), C18: 0 (23.4%) and C18: 1n9 (9.0%) (17). In boar sperm, the most abundant saturated fatty acids (SFAs) were C16: 0 (18%) and C18: 0 (16%), and the most abundant FAs were DPA (15%) and docosahexaenoic acid (DHA) (16%) (18). PUFAs account for nearly 60% of the total FAs in mammalian sperm (19); in particular, testicular cells and sperm contain large amounts of PUFAs, which are considered to be the major constituents of sperm phospholipids (20). It can be seen that regulating the composition of sperm FAs of different species is of great significance for improving semen quality in the future.

This review will focus on the effects of the four most important FAs including SFAs, monounsaturated fatty acids (MUFAs), omega-3 PUFAs and omega-6 PUFAs on semen quality. Our aim is to analyze the effect of FAs in diets, semen and extender on semen quality, and to extensively explore the potential mechanism of regulating animal semen quality.

## 2. Effect of different FAs on semen quality

FAs are classified into SFAs, MUFAs, and PUFAs, and a key difference between them is their degree of unsaturation, with zero, one, or more double bonds, respectively (21). There are many nomenclature systems for FAs, International Union of Pure and Applied Chemistry (IUPAC) can technically and clearly describe the chemical structure of FAs, but its name is too long. For convenience, historical names and shorthand notation are frequently used in scientific writings. The members of the common FAs families are shown in Table 1 (4).

**Table 1:** Abbreviations and sources of FAs.

Common nouns	Systematic name	Abbreviation	Sources
SFAs			
Myristic acid	Tetradecanoic	C14:0 (MA)	Dairy fat, coconut oil, palm kernel oil
Palmitic acid	Hexadecanoic	C16:0 (PA)	Most fats and oils
Stearic acid	Octadecanoic	C18:0 (SA)	Most fats and oils
MUFAs			
Oleic acid	cis-9-octadecenoic	9C-18:1 (OA)	All fats and oils, especially olive oil, canola oil and high-oleic sunflower and safflower oil
Palmitoleic acid	cis-9-hexadecenoic	9C-16:1 (PTA)	Marine oils, macadamia oil, most animal and vegetable oils
Omega-6 PUFAs			
Arachidonic acid	cis-5,cis-8,cis-11,cis-14-eicosatetraenoic acid	20∶4 (AA)	Animal fat, liver,egg lipids, fish
Linoleic acid	cis-9,cis-12-octadecadienoic	18∶2 (LA)	Most vegetable oils, nuts
Docosapentaenoic acid	cis-4,cis-7,cis-10,cis-13,cis-16-docosapentaenoic acid	22∶5 (DPA)	Fish (salmon, herring, anchovies, and mackerel)
Omega-3 PUFAs			
α-linolenic acid	cis-9,cis-12-cis-15-octadecatrienoic acid	18:3 (ALA)	Flaxseed, perilla, walnut, hemp seed, rapeseed, soybean
Docosahexaenoic acid	cis-4,cis-7,cis-10,cis-13,cis-16,cis-19-docosapentaenoic acid	22:6 (DHA)	Fish (salmon, herring, anchovy, smelt, and mackerel)
Eicosapentaenoic acid	cis-5,cis-8,cis-11,cis-14,cis-17-eicosapentaenoic acid	20:5 (EPA)	Fish (salmon, herring, anchovy, smelt, and mackerel)

SFAs include three types, PA, SA and myristic acid (MA). It has been reported that SFAs can improve sperm viability and plasma membrane integrity by enhancing sperm antioxidant activity, which may be beneficial for improving semen quality (22). However, another study pointed out that the percentage of SFAs in semen is inversely correlated with semen quality (23), and sperm motility and viability decrease when the ratio of SFAs in sperm increases (24). Therefore, it is speculated that the effect of SFAs on semen quality needs further research. PA is the major SFAs in sperm (25, 26), and its level in sperm is positively correlated with the total sperm count, indicating its importance for sperm production (27). SA is the predominant SFAs in many kinds of animal sperm, and is related to sperm plasma membrane integrity and oxidative stress (28). It has been reported that the content of SA may be affected by species factors (29). MA is a straight-chain SFAs with 14 carbon atoms and no carbon–carbon double bond, which was originally found in the seeds of nutmeg (30, 31). MA is also widely found in animal fats and vegetable oils, including sperm, whale oil, coconut oil, and dairy products (32, 33). Compared with other SFAs, MA exhibits higher antioxidant activity (34). Other studies have shown that MA can promote the interconversion of different FAs, thereby improving their availability (35).

In mammalian biological systems, MUFAs are the best indicator for assessing their role in membrane fluidity, as observed in sperm (20). MUFAs partially offset the negative effects of high-fat diet on sperm quality by increasing gamete motility, improving mitochondrial respiration efficiency, and reducing oxidative stress (36). Oleic acid (OA) is a MUFAs with 18 carbon chains belonging to the omega-9 family, and the antioxidant potency of OA has been widely recognized (37). Palmitoleic acid (PTA) is a MUFA with 16 carbon chains, which belongs to the omega-7 MUFAs. PTA is a natural ingredient in macadamia oil, sea buckthorn oil, and fish oil (38). Similar to the function of OA, the beneficial effects of PTA on antioxidant enzyme activity and altered signal transduction have been demonstrated in different cells (39). But another study showed that MUFAs were negatively correlated with sperm motility and concentration (22). It can be seen that the paradoxical effects of MUFAs on semen quality require further research.

**Figure 2:**
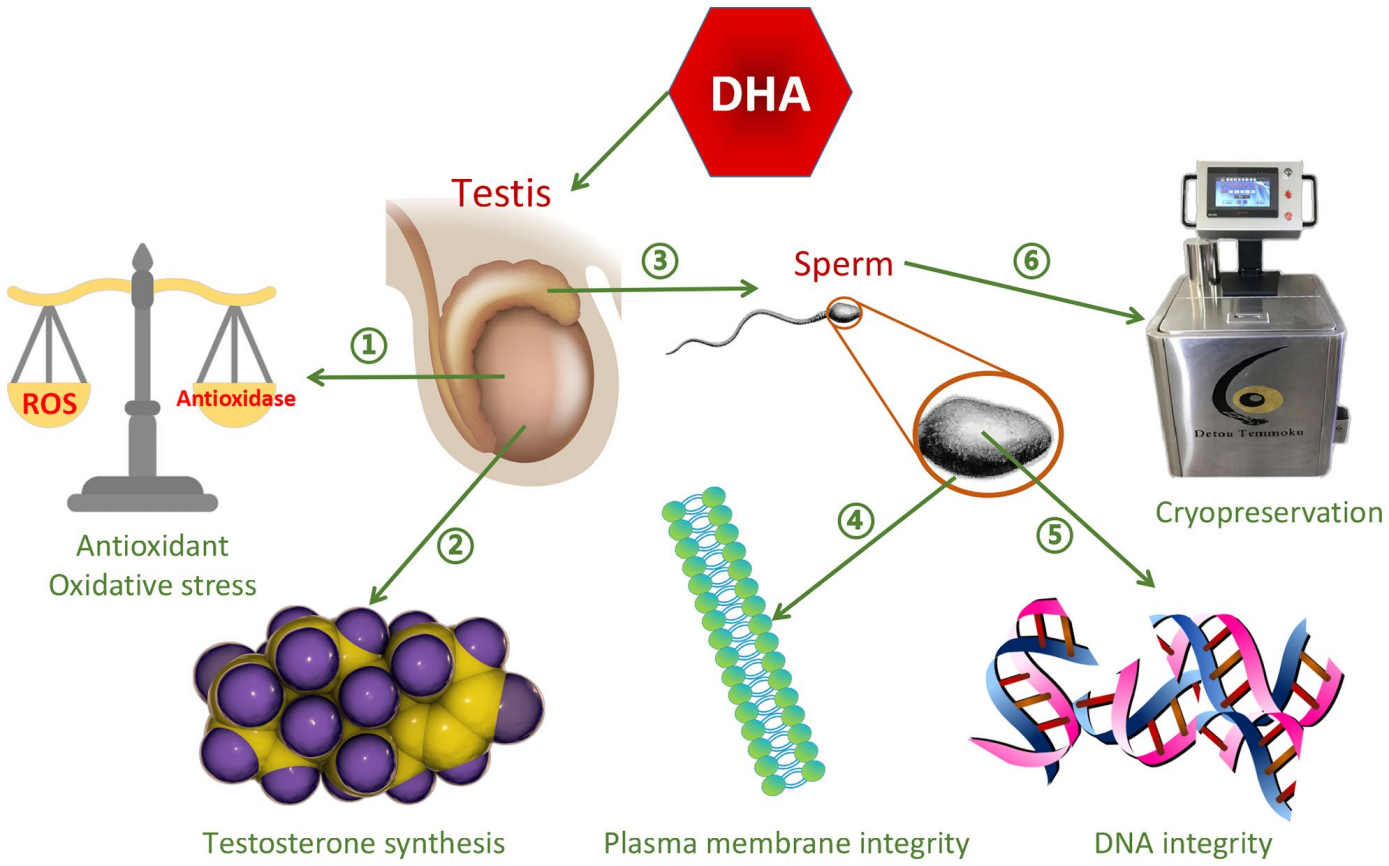
The function of DHA on semen quality. ➀Improve antioxidant capacity and reduce oxidative stress ②Promote testosterone synthesis ③Promote sperm maturation ④Improve sperm plasma membrane integrity ➄Improve DNA integrity ⑥Protect sperm from damage caused by cryopreservation.

Omega-3 PUFAs contain the first double bond at the third carbon atom at the methyl end of the FAs, and include three types, alpha-linolenic acid (ALA), Eicosapentaenoic acid (EPA), and DHA (40). Omega-3 PUFAs play important roles in reproductive physiology, including regulation of prostaglandin synthesis and membrane properties, and have excellent antioxidant effects (41). They are nutrients that improve semen quality (42), and accelerate spermatogenesis in different types of livestock (43). The most abundant omega-3 PUFAs is ALA (44), which has anti-inflammatory (45, 46) and antioxidant properties (47), while stimulating testosterone production (48). The six double bonds of DHA contribute to the maintenance of plasma membrane integrity (49), thereby providing membrane with very unique fluidity and flexibility, and thus are essential for improving sperm motility and acrosome reaction (50). In addition, DHA plays a positive role in promoting testicular testosterone secretion, improving sperm antioxidant capacity and DNA integrity (51). The function of DHA on semen quality is shown in Figure 2. Similar to the function of DHA, EPA is particularly important for sperm motility, normal morphology, plasma membrane integrity and freezing resistance (52). EPA is a precursor of eicosanoids such as prostaglandins, cyclic prostaglandins, thromboxane, and leukotrienes. Sperm lipids contain many different sphingolipids, among which sphingomyelin is rich in EPA, which plays an important role in regulating sperm motility and sperm plasma membrane structure (53). It can be concluded that different types of omega-3 PUFAs can improve semen quality.

It is well known that the Western diet is relatively poor in omega-3 PUFAs and rich in omega-6 PUFAs. Omega-6 PUFAs are essential FAs in vertebrates, including linoleic acid (LA), arachidonic acid (AA), and DPA, have been shown to regulate testicular function (54). AA has the effect of reducing inflammation (55), promoting phospholipid synthesis and secretion of various steroid hormones (56). The most plentiful dietary omega-6 PUFAs is LA (57). The sperm cell membrane of birds contains a large amount of LA, which is an important precursor for the synthesis of other omega-6 PUFAs (58). Conjugated linoleic acid (CLA) is a geometric isomer of LA that is mainly synthesized by bacteria in the rumen, which can regulate the synthesis of testosterone and improve the antioxidant capacity of sperm (59). DPA is present at low level in most organisms, whereas the cell membranes of mammalian brain and testis are rich in DPA (>3–4 times higher than other tissues) (60). It has been proposed that DPA is actively produced during sperm maturation in epididymal, and the change of its content in sperm membrane could affect sperm motility and viability, as well as sperm fertilization ability (61). In conclusion, different types of omega-6 PUFAs have different contents and functions, which need more exploration.

## 3. Effects of diets FAs on semen quality

Some studies have pointed out that the intake of high doses of SFAs poses risks to health, increases the level of oxidative stress, and reduces the synthesis of testosterone key enzymes, which will have adverse effects on semen quality (62, 63). Jensen et al. reported in a sample of 701 young Danish men that excessive SFAs intake resulted in a decrease in total sperm count and sperm concentration (64). In addition, Takato et al. (33) reported that long-term oral MA is beneficial to the human body, but the daily safe dietary dose should be less than 37.0 mg. Taken together, in order to avoid sperm damage, SFAs intake should be controlled within the minimum range.

Omega-3 PUFAs could improve sperm plasma membrane fluidity by trapping free radicals (65). When bulls were fed a diet rich in omega-3 PUFAs, the altered FAs profile in the sperm plasma membrane made the membrane more resistant to damage caused by ice crystal formation during freezing (66). Adding appropriate levels of omega-3 PUFAs to the diet has been reported to increase concentrations of IGF1, plasma testosterone, and scrotal circumference in male buffalo (67), and by increasing motility, viability, plasma membrane integrity, and acrosome integrity (68), reducing sperm lipid peroxidation to improve sperm quality and *in vitro* fertilization capacity (69). However, contrary results were previously reported, with a diet rich in omega-3 PUFAs making boar sperm more susceptible to lipid peroxidative damage (70), which negatively affected membrane structure and function (71). These conflicting results appear to be largely attributable to several factors, including age, breed, and source of omega-3 PUFAs (68). The ALA content of flaxseed oil reaches more than 50%, and studies have shown that supplementation of flaxseed oil in bull diets could have a positive effect on the progressive motility, morphology and viability of frozen–thawed sperm (72, 73). In the bulls supplemented with dietary DHA, although no improvement was found in the frozen–thawed semen, the viability of fresh sperm was improved (74). In a clinical trial, After 10 weeks of dietary DHA treatment, its content in seminal plasma was increased, antioxidant capacity was improved, and the percentage of sperm with DNA damage was reduced (51). In conclusion, dietary DHA supplementation increased its concentration in seminal plasma, which was associated with increased total antioxidant capacity and decreased sperm DNA fragmentation. Higher concentrations of EPA in the diet improved ejaculate volume and sperm motility (75). Supplementation of EPA in the diets can improve sperm motility, viability, total sperm count and total morphologically normal sperm count in dogs, as well as increase serum testosterone concentrations (43). It can be seen that EPA in the body is related to sperm motility and spermatogenesis.

It has been established that increasing dietary omega-6 PUFAs increases plasma steroid hormone levels (76). Other studies have shown that a diet rich in omega-6 PUFAs having a positive effect on semen quality and total sperm count in rams (77, 78), However, excess omega-6 PUFAs also expose sperm to oxidative stress damage (79). Supplementation of omega-6 PUFAs in bull diets can lead to lipid peroxidation, loss of PUFAs in the plasma membrane and reduced sperm motility and fertility (80). High levels of omega-6 PUFAs can lead to lipid peroxidation to produce malondialdehyde (MDA), which in turn inhibits sperm mitochondrial function and enzymatic activity, reducing DNA integrity and sperm motility (69). Therefore, when adding omega-6 PUFAs to diet, it is necessary to pay attention to its effect on sperm oxidative stress. It has been suggested that in ruminants, rumen microbes may hinder the transfer of dietary LA to semen because they hydrogenate LA (81). However, recent studies have shown that feeding additional LA results in significantly increased concentrations of LA in goat plasma and fertility in sperm, indicating that it has the ability to resist rumen biohydrogenation (82). Similarly, supplementation of CLA in the diets improved reproductive performance in dairy cows (83). When CLA was added at 50 g/day, there was a benefit on fresh and frozen–thawed sperm quality (84). However, 1% CLA supplementation in Japanese quail diets has been reported to reduce fertilization and hatchability (85), and CLA is thought to reduce spermatogenesis in rabbits and affect the synthesis of hormones involved in reproduction by affecting the composition of FAs in epididymal fat (86). Conflicting results may be caused by different species and ways of addition.

The predominant FAs in poultry sperm have been documented to be omega-6 PUFAs, whereas in most mammals omega-3 PUFAs are the predominant FAs (87). The lipids of sperm are relatively unique because they contain many different sphingolipids, including sphingolipids with DHA, EPA, and AA (88), and changes in lipid composition affect membrane fluidity, impair membrane function, and may even lead to intracellular death and apoptosis (89). The composition of sperm PUFAs has previously been reported to vary by diet, with the FAs content in sperm reflecting the ratio of omega-6/omega-3 PUFAs in the diet, and intake of different types and sources of PUFAs has been shown to alter sperm production in animals, while the composition of FAs in sperm could influence sperm quality, lipid composition, acrosome and fertilization ability (18).

**Figure 3:**
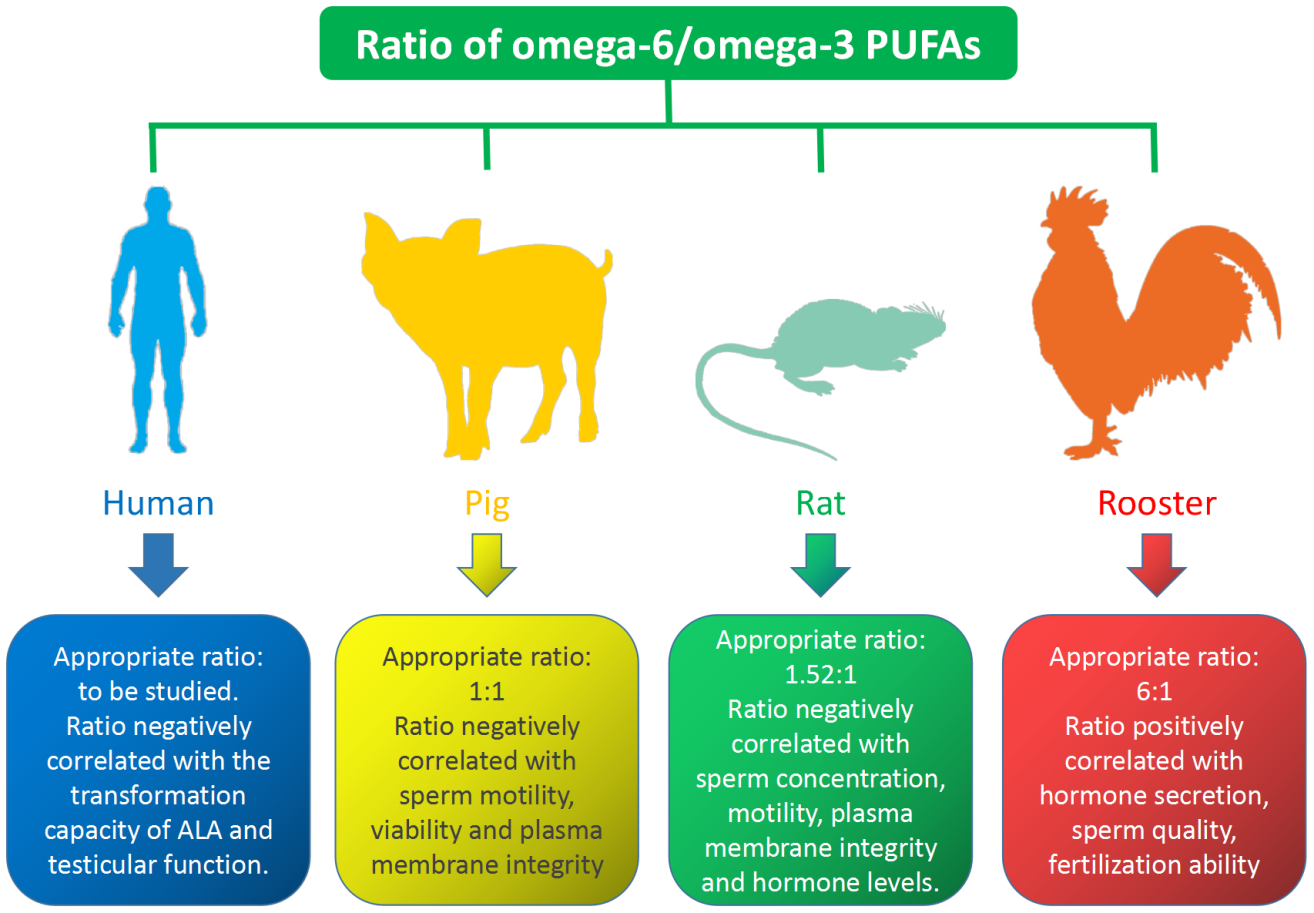
Effects of omega-6/omega-3 PUFAs on semen quality.

Numerous studies have shown that the omega-6/omega-3 PUFAs ratio has an important regulatory effect on male reproduction, the properties of which vary from species to species. The effect of the ratio of omega-6/omega-3 PUFAs on different species is shown in Figure 3. In the human diet, it is reasonable to maintain an appropriate dietary intake ratio of omega-6 and omega-3 PUFAs to promote reproduction. However, little is known about the effects of varying ratios of omega-6 and omega-3 PUFAs on sperm quality and fertility. Studies have shown that testicular function appears to be positively correlated with omega-3 PUFAs intake and negatively correlated with omega-6 PUFAs intake (90). Gerster found that the conversion of ALA to EPA and DHA was reduced by 40–50% when the ratio of omega-6/omega-3 PUFAs in the diet was elevated (91). Due to lack of research data, the appropriate ratio of omega-6/omega-3 PUFAs requires further research. Reducing the omega-6/omega-3 PUFAs ratio could improves boar plasma membrane properties and affects sperm FAs composition. Am-in N stated that the ratio of omega-6/omega-3 PUFAs in boar sperm was positively correlated with sperm motility, viability, normal morphology and normal plasma membrane (92). Appropriate omega-6/omega-3 PUFAs ratios in boar diets play an important role in maintaining boar reproductive performance, with an ideal ratio of 1: 1 (93). Similarly, increasing the ratio of omega-6/omega-6 PUFAs in the rat diets could decrease the concentrations of hormones such as GnRH, FSH, LH, and testosterone, while improving sperm concentration, motility, and plasma membrane integrity (94). The ideal ratio of omega-6/omega-3 PUFAs in the diet is 1.52: 1, however, when it is higher than 1.52:1, it would lead to a decrease in hormone levels, which in turn affects reproductive performance (7). In conclusion, there is a strong relationship between the omega-3/omega-6 PUFAs ratio and steroid hormone levels, which in turn regulate semen quality in rats. Avian semen is characterized by a relatively high proportion of omega-6 PUFAs (95). The sperm of male broiler breeders are rich in AA and DHA, and the fertility of male broiler breeder sperm is positively correlated with their ratio (96). A diet with a moderate ratio of omega-6/omega-3 PUFAs is beneficial for semen quality and reproductive outcomes in rooster. Dietary treatment of roosters with an appropriate omega-6/omega-3 PUFAs ratio increases hormone secretion, thereby improving sperm quality, Diets with omega-6/omega-3 ratios ranging from 6: 1 to 9: 1 improved sperm fertilization capacity (97). This is consistent with research conducted in aged roosters that a ratio of 6.25: 1 omega-6/omega-3 PUFAs dietary supplements is the optimal concentration for improved semen quality and reproductive performance in aged roosters, while a ratio below 6.25: 1 may increase the degree of sperm lipid peroxidation and reduced reproductive outcomes (98).

## 4. Effect of FAs in extender on semen quality

Previous research has shown that adding 75 μmol/L PA to boar extender improved sperm motility parameters, membrane integrity and acrosome integrity, and decreased sperm apoptosis rate (45, 46). Similarly, it was found that the addition of PA at concentrations of 10–100 μmol/L to the bull extender significantly increased progressive linear motion, viability, and SOD levels, while reducing reactive oxygen species (ROS) levels on days 1 and 3 of the experiment (14). It can be seen that the addition of PA to the extender can improve the quality of semen, but the optimal dose may vary depending on the species.

The protective effect of OA on frozen sperm is reflected in improving its antioxidant capacity. The addition of OA at 0.125, 0.25, 0.5 and 1 mmol/L to the rooster extender increased the total antioxidant activity and decreased sperm MDA concentrations in seminal plasma stored at 4°C for 24 and 48 h, while the concentration of 1 mmol/L OA improves sperm motility in roosters (15). Similarly, adding 1.25 mmol/L OA to the extender and incubating at 37°C for 4 h significantly improved acrosome integrity, motility and viability of boar sperm (99). Addition of 0.25 and 0.50 mmol/L OA resulted in decreased lipid peroxidation and increased total antioxidant capacity, while improving motion parameters of frozen–thawed ram sperm. OA positively affects ram sperm plasma membrane integrity, motility, and stability during cold storage, increasing SOD activity after 48 h of storage at low temperature (100). In conclusion, the addition of OA to the extender may have a positive effect on semen quality. Compared to other natural FAs, PTA is the most reliable protective compound during sperm freezing, although it is in far lower levels in sperm phospholipids than other natural FAs (101). Studies have shown that 1 mmol/L PTA could increase the percentage of sperm motility in roosters stored at 4°C, and improving semen quality by increasing the activity of total antioxidant enzymes and reducing lipid peroxidationin seminal plasma after 24 and 48 h of storage (102). Similar to the results, 0.5 mmol/L PTA supplementation in ram extender increased the percentage of sperm forward motility, increased total antioxidant enzyme activity at 24, 48 and 72 h, and decreased sperm at 72 h storage of lipid peroxidation, when supplemented at concentrations of 0.25, 0.5 and 1 mmol/L PTA increased SOD activity in sperm and decreased NO production in sperm at 48 and 72 h during refrigeration (103). Addition of PTA to boar extender increased sperm viability and motility after 2 and 7 days of storage at 6°C, and increased sperm counts with active mitochondria after 3 days of storage (104). It is suggested that PTA can be added to the extender as an exogenous antioxidant. Taken together, different types of MUFAs in the extender can maintain the viability of frozen–thawed sperm by increasing the activity of total antioxidant enzymes, reducing lipid peroxidation.

At present, ALA has been studied for cryopreservation of buffalo semen, and the addition of 5 ng/mL ALA to the extender increased the omega-3 PUFAs content of the plasma membrane of the sperm head and tail, thereby improving the fluidity of the plasma membrane (105). The addition of ALA to the extender showed improvements in frozen–thawed sperm motility, progressive motility, plasma membrane integrity, viability, and chromatin integrity of buffalo bull sperm (106). In addition, another study reported that DHA supplementation in extender protected sperm from damage caused by the cryopreservation process (22). It indicated that the addition of different types of Omega-3 PUFAs in the extender may improve sperm antioxidant activity, reduce oxidative stress, and further maintain the plasma membrane integrity of frozen sperm.

It has been reported that addition of 0.25 mmol/L LA to the extender increased sperm motility and percentage of progressive motility in rooster (107). Addition of LA to the extender can improve the motility parameters, DNA integrity, plasma membrane integrity and reduce sperm oxidative stress damage of bull sperm after frozen–thawed (108). Similarly, CLA is a potent antioxidant that reduces lipid peroxidation induced by cryopreservation of boar sperm, enables long-term survival of sperm after refrigeration at 17°C (109, 110), and enhances the protective effect of sperm cryopreservation (111). Therefore, it is speculated that different types of LA can improve sperm quality after frozen–thawed by reducing oxidative stress damage.

## 5. FAs content affects semen quality

PA is one of the most abundant long-chain SFAs in rooster sperm. Similar results were found in mammals, the most abundant SFAs in the plasma membrane of Norwegian Landrace and Duroc varieties was PA, which was positively correlated with sperm survival and plasma membrane integrity (18). Further research indicates that that PA content in sperm is a key indicator for screening bulls for high or low fertility phenotypes (112). However, studies on human sperm showed that the level of PA in semen of infertile men and asthenospermia patients was higher than that of normal sperm (27, 113). And the increase of PA level leads to the disorder of sperm plasma membrane metabolism (114). The reason for the different results may be caused by different species. Unlike rooster sperm, the major SFAs of all major classes of buffalo sperm and seminal plasma is SA (115). Similarly, biochemical analysis of sperm lipids from three pterosaur species revealed that SA is the predominant SFAs in flying-fox sperm (116). Compared with silver fox sperm, blue fox sperm membranes had significantly higher SA content (29). Studies have shown that SA is a biomarker for identifying high freezability and low freezability male donkey sperm, which is related to the plasma membrane integrity and oxidative stress of sperm after frozen–thawed (28). However, another study reported a negative correlation between SA content and sperm motility in semen samples from 155 patients when tested by gas chromatography (117). This indicated that there were species differences in the effect of SA on semen quality.

DHA content increases during sperm epididymal maturation, and its deficiency in sperm is typical of male infertility or infertility (118). DHA content is positively correlated with sperm concentration and motility, and has a protective effect on DNA fragmentation (26). It is possible to affirm that increased sperm DHA concentration is essential for the final step in dog epididymal maturation, as it is directly involved in the events required for fertilization (119). DHA is extremely abundant in male ejaculate, and its content in sperm accounts for 44.9% of PUFAs (22) and 31.5% in seminal plasma of all the FAs (6). In bulls, DHA is approximately 30% in sperm and 20% in seminal plasma of total FAs (120). Safarinejad reported a positive correlation between EPA levels and semen quality in oligoasthenospermia men (121). Further study showed that EPA can improve sperm motility, but does not affect sperm concentration or sperm PUFAs content (18). It can be seen that the content of omega-3 PUFAs in sperm is rich, and its content is positively correlated with semen quality.

It was found in omega-6 PUFAs studies, AA in rat testis could regulate spermatogenesis and androgenic activity, and increase plasma FSH, LH, and testosterone levels in a dose-and time-dependent manner (122). *In vitro* study has also shown that the peroxygen product of AA causes the release of LH and FSH from porcine anterior pituitary cells (123). In summary, AA has an important regulatory effect on the synthesis of steroid hormones. The concentration of AA is associated with resistance to heat shock in boar sperm (124), and is positively correlated with deer semen quality (125). The above studies indicate that AA is an important omega-6 PUFAs regulating mammalian semen quality. Testis and sperm have a characteristic lipid composition, and high DPA concentrations are unique to rat testis, and its level was found to be independent of the quality and quantity of oil supplemented in the diet of mature rats (126). Although the source of DPA accumulation during early growth has not been elucidated, current studies have shown that PUFAs are transferred from the circulatory system to the liver and synthesize DPA, which eventually accumulates in the testis (127).

## 6. Mecanisms involved in FAs impacts on semen quality

**Figure 4:**
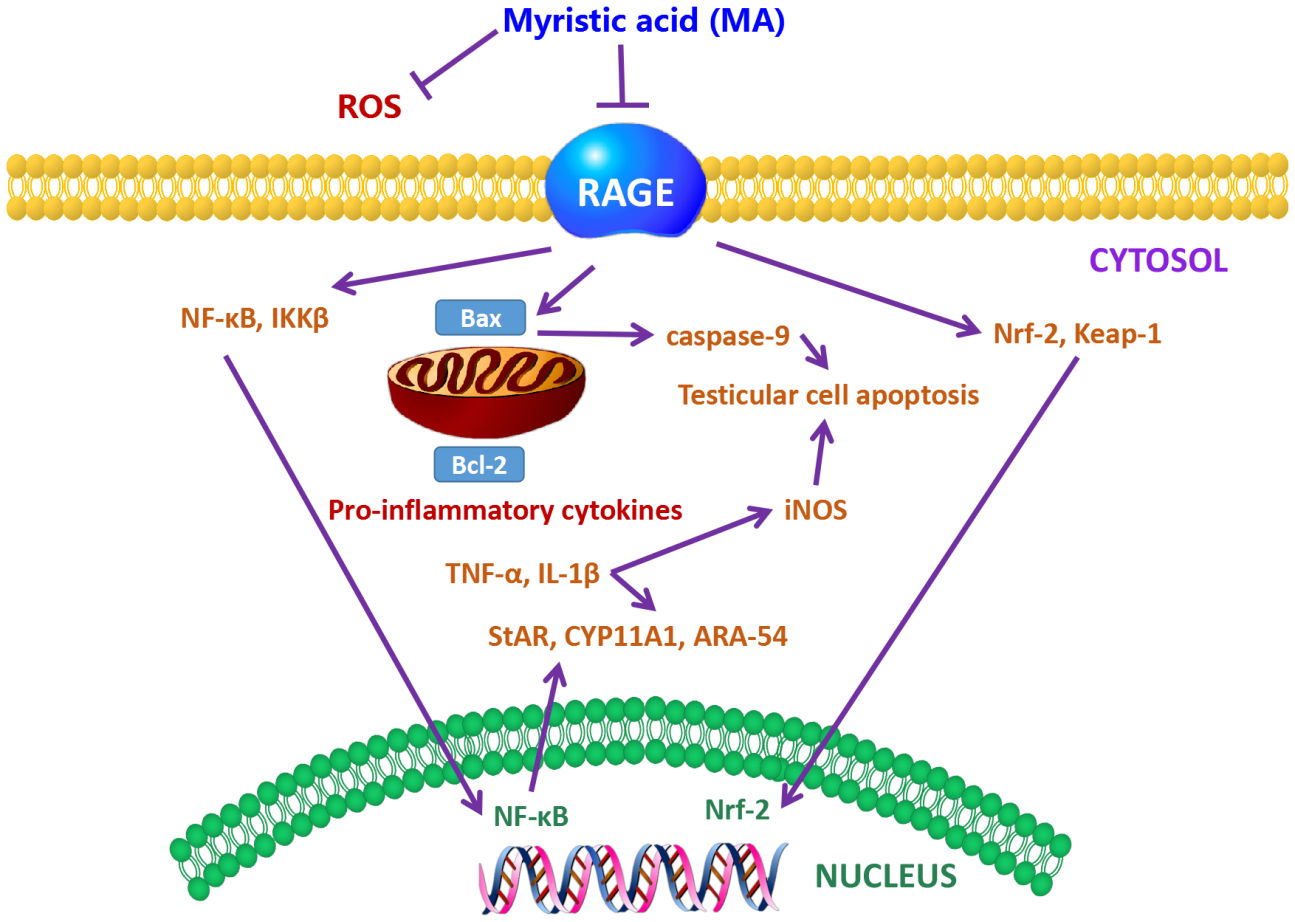
The protective mechanism of MA on sperm (128). Schematic showing the mechanism of MA protection in the testis. MA may lead to the down-regulation of RAGE and the reduction of ROS levels. Meanwhile, MA may also prevent the increase of lipid peroxidation and antioxidant enzymes by reducing the alteration of Nrf2-Keap1 pathway. MA treatment has also been postulated to have the ability to inhibit the NF-kβ pathway, resulting in the down-regulation of NF-kβ and IKKB, and thus possibly the reduction of TNF-α, IL-1β, and iNOS levels. In addition, MA can also prevent apoptosis by down-regulating the expression of Bax. MA treatment helps to increase the levels of steroidogenic markers such as StAR, CYP11A1 and ARA-54 in the testis of DM.

It has been reported that MA prevented the down-regulation of testicular steroidogenesis gene expression, and inhibited the reduction of sperm count, motility, viability and number of sperm with abnormal morphology in diabetic rats (128). Furthermore, oral administration of 10 and 20 mg/kg body weight of MA to rats for 28 consecutive days also prevented the increase in testicular inflammation and apoptosis by preventing the down-regulation of oxidative stress-related genes (129). Further study indicates that MA treatment reduced MDA and ROS levels in the testis of diabetic rats, and decreased receptor for advanced glycation endproducts (RAGE) up-regulation, thereby protecting the testis from oxidative damage and maintaining sperm motility and sperm morphology (130). The protection mechanism of MA is shown in Figure 4. Taken together, MA has a protective effect on testicular oxidative damage, thereby helping to prevent the occurrence of testicular inflammation and apoptosis. Sperm can use PA as a substrate to generate more ATP as an energy source for maintaining viability (131). However, other studies have shown that PA increases glycolytic flux and lactate production in testicular tissue cells *in vitro*, as well as carnitine palmitoyltransferase I (CPT1) and long-chain acyl-CoA dehydrogenase (LCAD) levels, These two enzymes are key enzymes in the β-oxidation of FAs. Meanwhile, mitochondrial respiration was impaired by PA followed by decreased ATP turnover, increased maximal respiration, and proton leak (132). The different results may be caused by species difference and different PA content in sperm, which need further study.

It was found that ALA is the parent of the omega-3 PUFAs, which mammals convert to EPA and DHA through alternating elongation and desaturation by elongases of very long chain FAs (ELOVL) and fatty acid dehydrogenase (FADs) enzymes (133, 134). It was found in a further study that the liver can synthesize EPA, and its precursors are transported to the testis with the blood to improve sperm motility. There is a negative correlation between EPA in the liver and sperm production, indicating that it is synthesized in the liver and then transferred to the testis to promote the production of sperm plasma membrane (18). It shows that different types of omega-3 PUFAs play an important role in maintaining the content of different types of FAs in the body, and the liver may be an important place for synthesizing FAs.

**Figure 5:**
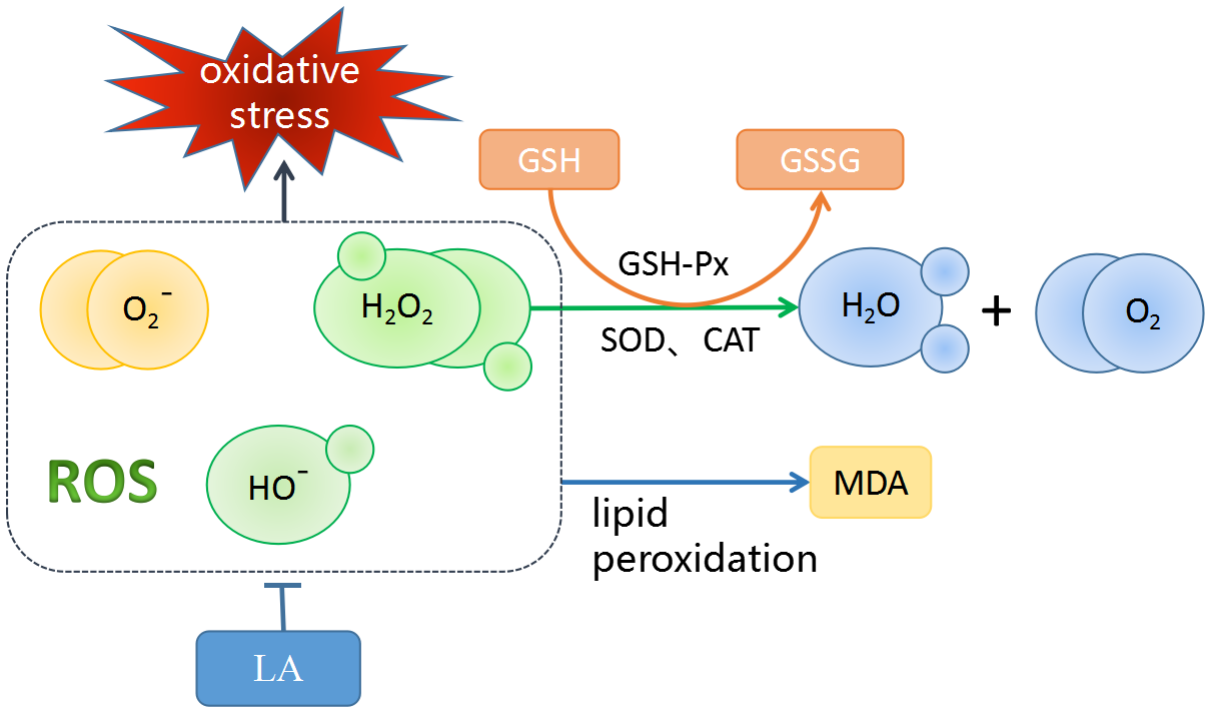
Antioxidant Mechanisms of LA. LA could scavenge ROS that play an important role in the initiation of lipid peroxidation. MDA can be used as an indirect measure of accumulated lipid peroxidation. Glutathione peroxidase (GSH-Px) is an enzyme that oxidizes reduced glutathione (GSH) to oxidized glutathione (GSSG), a process that reduces lipid peroxides to the corresponding alcohol, and reduces free hydrogen peroxide to water and molecular oxygen. Meanwhile, superoxide dismutase (SOD) and catalase (CAT) also have similar functions.

AA is part of the cell–cell signaling regulatory network for spermatogenesis (135). A small amount of AA does not cause oxidative stress damage to sperm (136). However, excessive AA activates lipoxygenase (LOX) and mitogen-activated protein kinase (MAPK) signaling pathways (137), increasing sperm oxidative damage and reducing sperm motility in a dose-dependent manner (1, 138). Therefore, it can be speculated that an appropriate dose of AA can improve semen quality, but it is also important to avoid oxidative stress damage to sperm caused by AA. Unlike AA, LA can improve the antioxidant capacity of sperm by providing a suitable antioxidant/oxidative ratio (2, 139). Figure 5 shows the antioxidant mechanism of LA. As a geometric isomer of LA, CLA can be converted to other PUFAs for the needs of the organism through a series of elongation and desaturation steps performed by different enzymes in the endoplasmic reticulum (140). In homeostasis, DPA, as the major PUFAs in the testis, is thought to be involved in the function of sperm transport, and the testis supports normal spermatogenesis by consuming DPA to transport sperm into the seminal vesicles (141). DPA is beneficial for the maintenance of sperm quality, is mainly stored in the testis as phospholipids containing PUFAs, increases the activity of endogenous antioxidant enzymes to maintain the DNA integrity of sperm in the testis (142).

## 7. Summary

The beneficial and detrimental effects of FAs supplementation in diets and extender are currently the focus of research in the field of male reproduction. In this review, we brought together recent findings the effects of different types of FAs on semen quality. The ratio of omega-6/omega-3 PUFAs in sperm is related to semen quality. To improve male fertility and for economic reasons, future studies should focus on analyzing the composition of sperm FAs and exploring its role in the regulation of extender and diets on semen quality. Our aim for a future in which FAs will help to improve the quality of animal semen production. It can be concluded that the FAs composition of diets and supplements affects sperm metabolism, and analysis of their profile in semen will be an important indicator for identifying sperm fertility. At this moment, FAs have varying effects on semen properties, which may be affected by its species, animal species, treatment methods and dosage. It is still too early to ensure optimal levels of FAs additions to diets or extender with a view to developing diets or extender that improve semen quality. Although some FAs have proven very promising, their efficacy and practical applicability need to be validated in future. Future studies should not only clarify the requirements of different animal for FAs, but also deeply explore the mechanism of its impact on semen quality, so as to develop diets or extender that can improve semen quality.

## Author contributions

CY: writing—original draft preparation. JW: writing—review and editing. WL: funding acquisition. All authors contributed to the article and approved the submitted version.

## Funding

This work was supported by the 14th Five-Year Key Research and Development Special Sub-project of the Ministry of Science and Technology (2021YFF1000701); Young and middle-aged scientific and technological innovation leaders and teams in Jilin Province (20200301031RQ); and the China Agriculture Research System of MOF and MARA (CARS-37).

## Conflict of interest

The authors declare that the research was conducted in the absence of any commercial or financial relationships that could be construed as a potential conflict of interest.

## Publisher’s note

All claims expressed in this article are solely those of the authors and do not necessarily represent those of their affiliated organizations, or those of the publisher, the editors and the reviewers. Any product that may be evaluated in this article, or claim that may be made by its manufacturer, is not guaranteed or endorsed by the publisher.
